# The Art of Capturing Pluripotency: Creating the Right Culture

**DOI:** 10.1016/j.stemcr.2017.05.020

**Published:** 2017-06-09

**Authors:** Qi-Long Ying, Austin Smith

**Affiliations:** 1 Eli and Edythe Broad Center for Regenerative Medicine and Stem Cell Research at USC, Department of Stem Cell Biology and Regenerative Medicine, Keck School of Medicine, University of Southern California, Los Angeles, CA 90033, USA; 2 Wellcome Trust – Medical Research Council Stem Cell Institute, University of Cambridge, Tennis Court Road, Cambridge CB2 1QR, UK; 3 Department of Biochemistry, University of Cambridge, Tennis Court Road, Cambridge CB2 1GA, UK

**Keywords:** embryonic stem cell, epiblast stem cell, naive pluripotency, primed pluripotency, stem cell self-renewal, stem cell differentiation, LIF/Stat3, Wnt/β-catenin, MEK, ERK

## Abstract

Embryonic stem cells (ESCs) are a unique tool for genetic perturbation of mammalian cellular and organismal processes additionally in humans offer unprecedented opportunities for disease modeling and cell therapy. Furthermore, ESCs are a powerful system for exploring the fundamental biology of pluripotency. Indeed understanding the control of self-renewal and differentiation is key to realizing the potential of ESCs. Building on previous observations, we found that mouse ESCs can be derived and maintained with high efficiency through insulation from differentiation cues combined with consolidation of an innate cell proliferation program. This finding of a pluripotent ground state has led to conceptual and practical advances, including the establishment of germline-competent ESCs from recalcitrant mouse strains and for the first time from the rat. Here, we summarize historical and recent progress in defining the signaling environment that supports self-renewal. We compare the contrasting requirements of two types of pluripotent stem cell, naive ESCs and primed post-implantation epiblast stem cells (EpiSCs), and consider the outstanding challenge of generating naive pluripotent stem cells from different mammals.

## Teaser

In this Perspective, Ying and Smith review the refinement of self-renewal conditions for mouse embryonic stem cells leading to the defined ground state culture system. The authors discuss the distinction between naive and primed pluripotency and consider the prospects for establishing ground state pluripotent stem cells from other mammals.

## Main Text

### Nurture of Pluripotent Embryonic Stem Cells

The study of pluripotency began with the discovery of a strain of mice that spontaneously developed teratocarcinoma (Stevens and Little, 1954). These multi-differentiated tumors comprise derivatives of all germ layers along with an undifferentiated proliferative compartment. The primitive proliferative cells, called embryonal carcinoma (EC) cells, could be propagated in culture and remain pluripotent (Martin, 1980). However, EC cells are karyotypically abnormal and tumorigenic (Silver et al., 1983). Fortunately, culture conditions optimized for EC cells subsequently allowed the derivation of pluripotent stem cells directly from pre-implantation mouse embryos (Evans and Kaufman, 1981, Martin, 1981). These embryonic stem cells (ESCs) were genetically normal and exhibited the remarkable capacity to contribute extensively to chimeric mice without forming tumors (Bradley et al., 1984). Moreover, ESCs could colonize the germline in chimaeras, heralding the era of targeted manipulation of the mouse genome (Capecchi, 2005).

**Figure:**
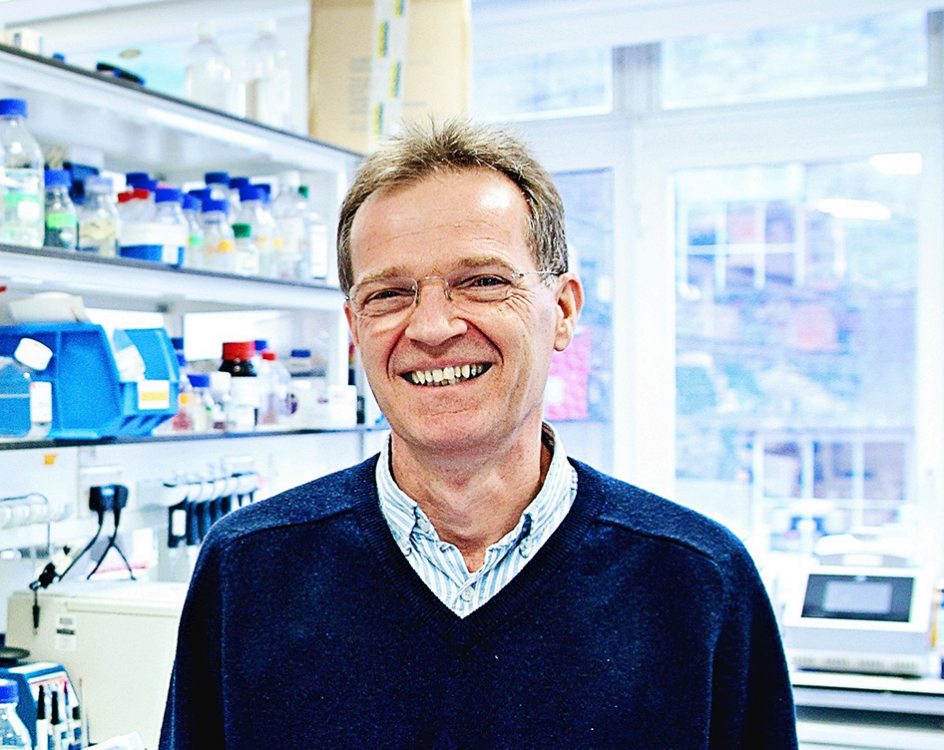
Austin Smith graduated in Biochemistry from the University of Oxford in 1982 and then pursued PhD studies in Developmental Genetics in Edinburgh. After a post-doctoral period back in Oxford, he returned to Edinburgh in 1990 as a Group Leader at the Centre for Genome Research. He became Centre Director in 1995 and formed the Institute for Stem Cell Research. In 2006 he moved to the University of Cambridge and was founding Director of the Cambridge Stem Cell Institute. His research has centred on embryonic stem cells. His main interest is to elucidate how the in vitro self-renewal and lineage specification of pluripotent stem cells relates to generic principles for establishment and capacitation of pluripotency in mammalian embryos. Austin Smith is a Medical Research Council Professor, a member of EMBO, and a fellow of the Royal Societies of Edinburgh and of London. In 2016 he was honoured to receive jointly with his former post-doctoral colleague, Ying Qi-Long, the ISSCR McEwen Award for Innovation.

ESCs were originally derived by co-culture with a feeder layer of mitotically inactivated fibroblasts in medium containing fetal calf serum. These empirical culture conditions are effective for deriving and propagating ESCs from the inbred 129 strain of mice, but less so or not at all for other strains. Eventually it was found that feeders could be replaced by the cytokine leukemia inhibitory factor (LIF) (Smith et al., 1988, Smith and Hooper, 1987, Williams et al., 1988) and that serum could be substituted by bone morphogenetic protein (BMP) (Ying et al., 2003). These findings provided a defined culture condition but did not enable generic derivation of ESCs from different mouse strains. nor did they maintain homogeneous cultures.

**Figure:**
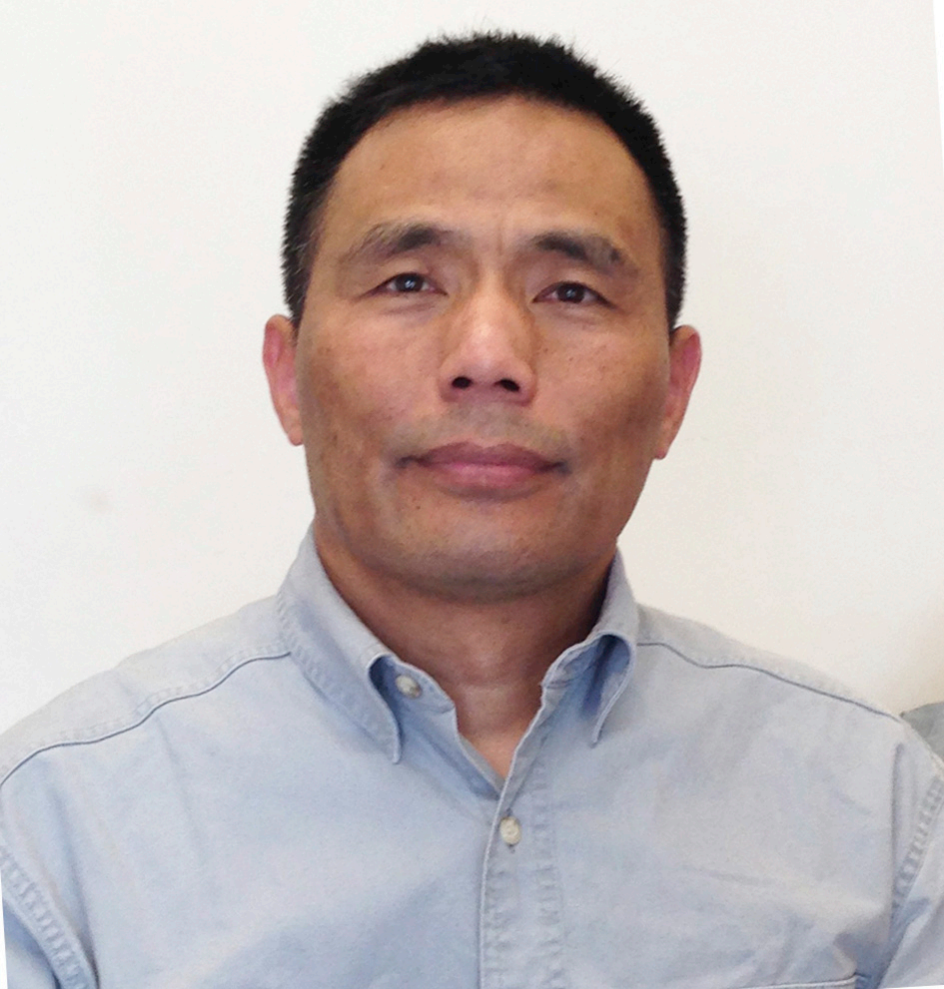
Dr. Qi-Long Ying is an Associate Professor of Stem Cell Biology at the Eli and Edythe Broad Center for Regenerative Medicine and Stem Cell Research, University of Southern California (USC). He obtained his bachelor's degree in medicine from the First Military Medical University (now Southern Medical University, China) in 1987. Qi-Long went on to earn MSc and Ph. D degrees from the Department of Neurosurgery, Huashan Hospital, Shanghai Medical University while also practicing clinical neurosurgery. From 1995-1998, he undertook his first postdoctoral study in Prof. Houyan Song’s laboratory in the Department of Molecular Genetics, Shanghai Medical University. He then joined Austin Smith’s group at the University of Edinburgh. During his years with Austin, Qi-Long made seminal findings that led to new culture systems for the propagation and differentiation of mouse embryonic stem cells. In 2006, Qi-Long moved to USC to set up his own laboratory. His current research focuses on understanding the molecular basis of embryonic and tissue-specific stem cell self-renewal. Qi-Long and Austin were honored with the McEwen Award for Innovation at the 2016 International Society for Stem Cell Research annual meeting in San Francisco, USA.

LIF signaling is not essential for pluripotency in vivo (Stewart et al., 1992), except during facultative diapause (Nichols et al., 2001). This observation suggested that it should be possible to bypass the requirement of ESCs for LIF. LIF supports ESC self-renewal through receptor-mediated stimulation of Janus-associated kinase (JAK) and activation of the transcription factor Stat3 (Niwa et al., 1998). LIF also activates the mitogen-activated protein kinase (Erk) cascade, but this is dispensable for self-renewal. To the contrary, we found that inhibition of Erk activation actually promoted the self-renewal response (Burdon et al., 1999). However, pharmacological or genetic suppression of Erk signaling was not sufficient to sustain ESCs long-term or clonally without LIF (Wray et al., 2010). We therefore investigated alternative signaling pathways.

**Figure 1:**
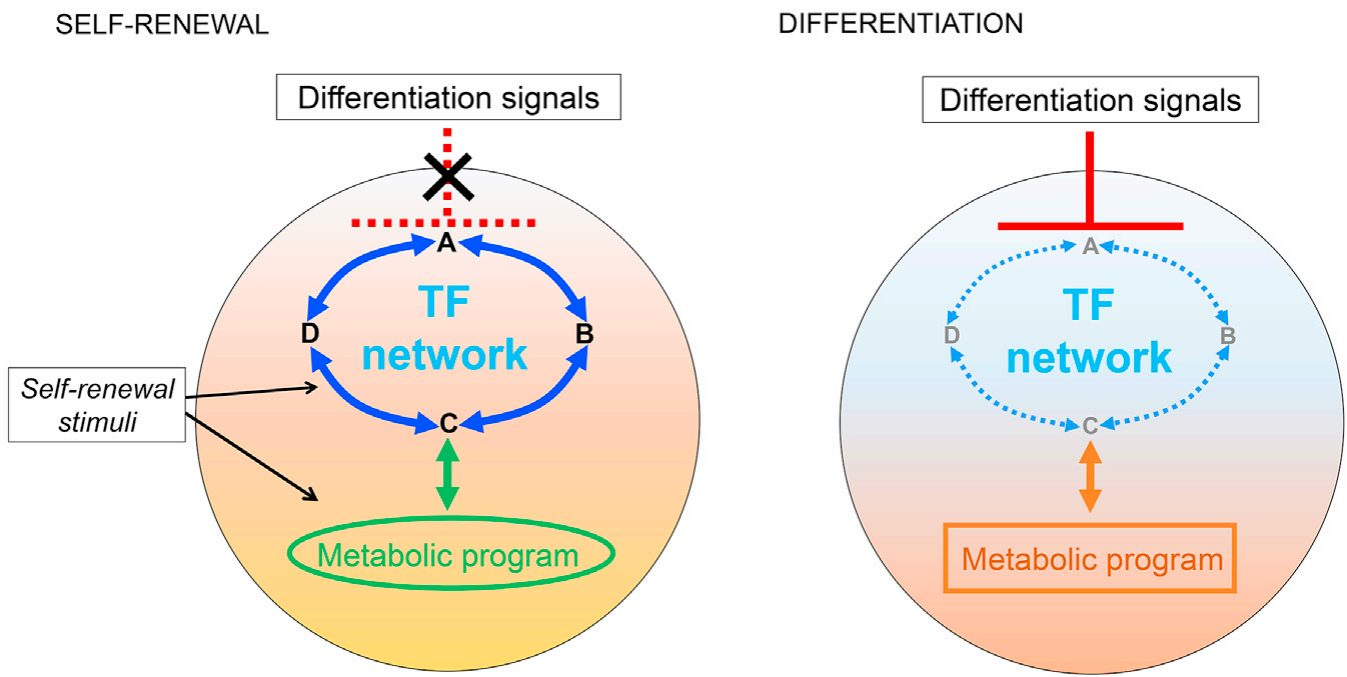
Capture of a Stem Cell State by Suspending Developmental Progression A generic scheme illustrating the idea that self-renewal can ensue if the core transcription factor (TF) network is insulated from differentiation cues and requisite metabolic conditions are satisfied. In the specific case of the mouse ESC ground state, the 2i inhibitors block FGF/Erk and Tcf3 differentiation pathways, while LIF boosts the core TF network and also promotes metabolic activity, as does GSK3 inhibition. In the absence of 2i/LIF components, the ESC gene regulatory circuitry collapses and cells transition toward lineage priming and differentiation.

It had been reported that simulation of Wnt signaling by inhibition of glycogen synthase kinase 3 (GSK3) could support ESC self-renewal (Sato et al., 2004). Interpretation of those studies was uncertain, however, because the indirubin class of GSK3 inhibitors displayed pleiotropic off-target effects and the available recombinant Wnt had a comparatively weak effect that appeared to rely on synergy with LIF (Hao et al., 2006, Ogawa et al., 2006). Following advice from Philip Cohen (University of Dundee), we obtained a more selective GSK3 inhibitor, CHIR99021 (CHIR) (Bain et al., 2007). Using titrated CHIR we found that partial inhibition of GSK3 had a short-term stimulatory effect on ESC self-renewal in the absence of LIF and serum. We then combined CHIR with blockade of the Erk pathway, initially using inhibitors of both the Erk activating enzymes MEK1/2, and of the fibroblast growth factor (FGF) receptor. This three-inhibitor combination (3i) was sufficient to derive and maintain mouse ESCs without LIF or serum and even upon genetic deletion of Stat3 (Ying et al., 2008). We subsequently noted that a more potent MEK inhibitor, PD0325901 (PD03) (Bain et al., 2007), rendered the FGF receptor inhibitor dispensable. The two-inhibitor (2i) combination has since been widely adopted. These two inhibitors effectively shut down differentiation pathways in naive cells while preserving their intrinsic metabolic and proliferative program (Figure 1).

Importantly, although 2i allows strain 129 ESC propagation independently of LIF/Stat3 activity, addition of LIF gives superior clonal propagation (Wray et al., 2010) and may be crucial for expansion in other strains. Indeed a key advance enabled by culture in 2i/LIF without serum is the ability to derive germline-competent ESCs from different mouse strains (Nichols et al., 2009) and even from a different species, the rat (Buehr et al., 2008, Li et al., 2008). Thus use of 2i/LIF revealed that ESC derivation is a generic feature in mice and rats and not an artifact of a specific inbred background. Furthermore, molecular reprogramming of mouse somatic cells into induced pluripotent stem cells (Takahashi and Yamanaka, 2006) is facilitated by 2i/LIF (Silva et al., 2008).

### Molecular Circuitry of Mouse ESC Self-Renewal

The POU homeodomain transcription factor Oct4 and the HMG-box transcription factor Sox2 are considered to lie at the heart of pluripotency gene regulatory networks (Niwa, 2014, Young, 2011). Strikingly, however, neither LIF nor 2i appear to regulate these factors directly. Research over several years has identified several transcription factor target genes of Stat3 in ESCs, including *Klf4*, *Tfcp2l1*, and *Gbx2* (Bourillot et al., 2009, Hall et al., 2009, Martello et al., 2013, Niwa et al., 2009, Tai and Ying, 2013, Ye et al., 2013, Ye et al., 2016). These three targets are each individually capable of mimicking the self-renewal-promoting effect of LIF when overexpressed. GSK3 inhibition also upregulates several transcription factors with self-renewal activity. GSK3 exists in two isoforms α and β, both of which are inhibited by CHIR. Treatment of ESCs with CHIR leads to induction of Esrrb, Nanog, and Tfcp2l1 (Martello et al., 2012, Ye et al., 2013). The mechanism has been debated, but genetic evidence indicates that critical mediators are β-catenin and Tcf3 (Guo et al., 2011, Lyashenko et al., 2011, Pereira et al., 2006, Wray et al., 2011, Ye et al., 2017). GSK3 inhibition stabilizes intracellular β-catenin, which is known to bind Tcf3 and relieve its repressive action at target genes (Shy et al., 2013, Wray et al., 2011, Yi et al., 2011). Thus the potent impact of LIF and CHIR on ESC self-renewal can largely be explained by combinatorial induction and derepression, respectively, of components of the pluripotency gene regulatory network (Dunn et al., 2014, Martello and Smith, 2014). In addition, however, LIF/Stat3 has pro-proliferative and metabolic effects that may be independent of induction of pluripotency factors (Carbognin et al., 2016). We have also noted that *β-catenin* null ESCs still respond to CHIR with a modest enhancement of self-renewal efficiency (Wray et al., 2011). GSK3 negatively regulates many targets other than β-catenin and acts broadly to suppress anabolic pathways (Doble and Woodgett, 2003). Therefore, inhibition of GSK3 can have a general growth-promoting effect, which may be particularly significant in a serum or growth factor-free environment and upon inhibition of Erk signaling.

The contribution of Erk pathway inhibition to ESC self-renewal also appears to be multi-factorial. There is evidence that active Erk can repress expression of Nanog (Hamazaki et al., 2006, Silva et al., 2009) and can inactivate Klf2 (Yeo et al., 2014). Therefore, Mek inhibition can increase the activity of these two self-renewal factors in the ESC network. However, Erk signaling may also actively promote developmental progression out of the ESC state (Kalkan and Smith, 2014, Kunath et al., 2007, Stavridis et al., 2007). Active Erk stimulates RNA polymerase, which may globally increase transcriptional noise as a basis for cell decision making, and/or be targeted to upregulate developmental genes (Tee et al., 2014). We additionally hypothesize, however, that Erk selectively induces or activates specific factors that mediate transition from the ESC state and installation of the succeeding gene regulatory network (Smith, 2017).

### Ground State or Metastability

Both 2i/LIF and serum/LIF support feeder-free self-renewal of ESCs competent for chimera formation and germline transmission after injection into blastocysts. The ESC populations in these two conditions are rather different, however. In serum/LIF the cultures are heterogeneous and expression of many early lineage genes is detectable (Marks et al., 2012). This is in part due to a degree of overt differentiation, but even within the Oct4-positive pluripotent compartment heterogeneity is apparent in morphology and gene expression. Most strikingly, many of the pluripotency transcription factors highlighted above, although not Oct4 or Sox2, exhibit fluctuating expression (Chambers et al., 2007, Filipczyk et al., 2015, Hayashi et al., 2008, Toyooka et al., 2008). Cells that have downregulated factors such as Nanog are more liable to differentiate (Torres-Padilla and Chambers, 2014) and tend to be excluded from chimeras (Alexandrova et al., 2016, Toyooka et al., 2008). These observations have been interpreted as reflecting an underlying metastability in pluripotent cells that provides an opportunity for lineage specification (Hayashi et al., 2008, Silva and Smith, 2008, Torres-Padilla and Chambers, 2014). Yet in 2i/LIF, ESCs display substantially uniform expression of pluripotency factors and negligible levels of most lineage-affiliated genes (Marks et al., 2012, Martello and Smith, 2014, Wray et al., 2010). Furthermore, mosaicism is not evident in the newly formed pluripotent embryonic epiblast between embryonic day 3.75 (E3.75) and E4.5 (Acampora et al., 2016, Boroviak et al., 2015). Thus metastability is not inherent to pluripotency. On the contrary, we have proposed that mouse ESCs in 2i/LIF occupy a ground state in which the pluripotency gene regulatory circuitry is maximally operative in all cells. This manifests as an equipotent and effectively homogeneous population of stem cells, exhibiting robust and sustained symmetric self-renewal.

### Naive and Primed Pluripotency

Pluripotency in the mouse embryo emerges in the mature inner cell mass and persists until the onset of somitogenesis, a period of around 5 days. Yet ESCs have only been derived directly from pre-implantation epiblast (Boroviak et al., 2014, Brook and Gardner, 1997, Evans and Kaufman, 1981). Post-implantation epiblast cells differentiate or die in any of the culture systems used for ESC propagation. Alternative conditions comprising stimulation with FGF and activin allowed derivation of pluripotent stem cells, termed EpiSCs, from post-implantation embryos (Brons et al., 2007, Tesar et al., 2007). EpiSCs are very different from ESCs transcriptomically, epigenetically, metabolically and functionally. They appear most related to the anterior primitive streak epiblast of the late gastrula (Kojima et al., 2014). Consistent with developmental trajectory, ESCs can be differentiated into EpiSCs by changing culture conditions whereas EpiSCs cannot, in general, revert to ESC status, except by transgenic expression of ESC transcription factors (Guo et al., 2009). The terms naive and primed were introduced to denote the early and late phases, respectively, of pluripotency in utero, and the corresponding ESC and EpiSC states in vitro (Nichols and Smith, 2009). Primed refers to the initiation of lineage specification in gastrula stage epiblast cells and EpiSCs, reflected in varying degrees of lineage-affiliated gene expression.

Significantly, the culture conditions applied to derive EpiSC were essentially the same as those used for propagating human pluripotent stem cells (Thomson et al., 1998, Vallier et al., 2005). The source of this and other differences between mouse ESCs and human pluripotent stem cells had long been debated, but with the arrival of EpiSCs the argument was largely settled; the major determinant is developmental stage rather than species (Rossant, 2015). This conclusion has been consolidated by the recent first molecular characterization of post-implantation epiblast in a non-human primate (Nakamura et al., 2016).

The primed status of human pluripotent stem cells may be a major contributing factor to the variability observed within and between different cell lines, which is also apparent in mouse EpiSCs but much less so in ESCs. Partly for this reason, efforts have been made to establish human naive stem cells. Simple application of 2i/LIF is not sufficient for human ESC derivation. Insufficiency of 2i/LIF may be in part because Esrrb, a key factor downstream of GSK3 inhibition in mouse ESCs, is not expressed in the human naive epiblast (Blakeley et al., 2015). A second difference is that the response to LIF/Stat3 signaling is much weaker in human than in mouse. Thus additional input may be essential to sustain the human naive pluripotency network in vitro. Recent reports indicate that supplementation of 2i/LIF with other pathway inhibitors can support propagation of human pluripotent stem cells with transcriptomic and epigenomic features of naive cells (Takashima et al., 2014, Theunissen et al., 2014), and enable their derivation directly from dissociated human inner cell mass cells (Guo et al., 2016). While there is scope to optimize the current culture systems, it seems likely that human equivalents of rodent ESCs are attainable, albeit with slightly different requirements.

### Pluripotent Plateaus

Mouse ESCs maintained in 2i/LIF or serum/LIF exhibit markedly different transcriptomes and epigenetic landscapes (Ficz et al., 2013, Habibi et al., 2013, Leitch et al., 2013, Marks et al., 2012). ESCs in 2i/LIF remain similar to E3.75–E4.5 pre-implantation epiblast from which they are derived (Boroviak et al., 2014, Boroviak et al., 2015, Brook and Gardner, 1997), whereas ESCs in serum/LIF diverge in both gene expression and DNA methylation. They remain distinct from EpiSCs, however. Notably, ESCs cultured in 2i/LIF or serum/LIF are readily interconvertible simply by switching culture conditions (Habibi et al., 2013, Joshi et al., 2015, Marks et al., 2012, Martin Gonzalez et al., 2016). Although some selection is apparent in the transition from serum, the efficiency of conversion within one passage implies a high degree of plasticity in ESCs. Similarly, aspects of the primed EpiSC phenotype adjust between different culture environments (Kim et al., 2013, Kurek et al., 2015, Sugimoto et al., 2015, Sumi et al., 2013, Tsakiridis et al., 2014, Wu et al., 2015). A “plateau model” has been proposed to explain such in vitro phenotype conversions (Chen et al., 2015). The plateau comprises stem cell populations captured in different culture conditions but representing a discrete phase of development. Particular conditions may maintain stem cells that diverge to a lesser or greater degree from the in vivo context. It is always important, therefore, to evaluate critically the resemblance of stem cells in a given culture context to resident embryo cells. Nonetheless, within the same plateau cells may readily be interconverted because developmental proximity can supersede distinctions imposed by culture environments. In contrast, conversion from the naive plateau to the primed plateau is unidirectional, and reversion can only be achieved efficiently via genetic or epigenetic manipulation.

A striking distinction between naive and primed pluripotent stem cells is their reliance on contrary signaling environments. Naive cells are characterized by self-renewal in the absence of FGF/Erk signaling and the presence of LIF/Stat3 stimulation, whereas primed cells require FGF/Erk and also stimulation of Smad2/3 by nodal, activin, or transforming growth factor β (Brons et al., 2007, Tesar et al., 2007, Vallier et al., 2005). Insulation of naive pluripotency from Erk appears crucial in restricting developmental transition and thereby sustaining self-renewal (Smith, 2017). A general feature of stem cell plateaus might be opposing extrinsic conditions to those required by differentiating progeny.

### Naive ESCs from Other Mammals

A key remaining challenge in the field of ESC biology is whether it is possible to establish authentic naive ESCs from species other than the rodent, and if so, how. Availability of ESCs from other species would extend the power of ESC-based genetic manipulation to large animal models and to livestock enhancement. Although there are many reports of embryo-derived cell cultures from different species, so far only mouse and rat ESCs have been validated by extensive contribution to adult chimeras after introduction into pre-implantation embryos.

It should be considered that naive pluripotency is a very transient phase of development, and self-renewal capacity is unlikely to be the direct target of evolutionary selection. However, we hypothesize that the fundamental program of pluripotency is conserved in mammals and that a core gene regulatory network capable of sustaining self-renewal may be retained. We suggest that the 2i culture system provides a paradigm for construction in vitro environments to sustain long-term stem cell self-renewal based on the principles of insulation from differentiation and reinforcement of requisite metabolic pathways (Figure 1). Variations in the 2i/LIF regimen have improved the derivation of rat ESCs (Chen et al., 2013) and have enabled the generation of candidate human naive pluripotent stem cells (Takashima et al., 2014, Theunissen et al., 2014) that show specific transcription factor expression related to the mouse ESC network (Dunn et al., 2014). Interestingly the requirement for GSK3 inhibition is diminished in rat and even more so in human, but resistance to abolition of Erk signaling is fully conserved. We anticipate that further refinements to prevent developmental progression entirely will enable capture of germline-competent naive ESCs from a broad range of mammals. We further speculate that identifying and suppressing signals that direct developmental transition may be a general approach to the capture of self-renewing stem cells.
